# Sharing Concerns: Interpersonal Worry Regulation in Romantic Couples

**DOI:** 10.1037/a0040112

**Published:** 2016-02-15

**Authors:** Brian Parkinson, Gwenda Simons, Karen Niven

**Affiliations:** 1 Department of Experimental Psychology, Oxford University; 2 Rheumatology Research Group, Institute of Inflammation and Ageing (IIA), University of Birmingham; 3 Manchester Business School, University of Manchester

**Keywords:** expressivity, close relationships, interpersonal emotion regulation, worry

## Abstract

Two dyadic studies investigated interpersonal worry regulation in heterosexual relationships. In Study 1, we video-recorded 40 romantic couples discussing shared concerns. Male partners’ worry positively predicted female partners’ interpersonal calming attempts, and negatively predicted female partners’ interpersonal alerting attempts (i.e., attempts to make their partners appreciate the seriousness of concerns). Video-cued recall data also indicated that changes in partner A’s worry over time positively predicted partner B’s motivation to reduce partner A’s worry, and that this effect was stronger when B was the female partner. Study 2 was a dyadic survey of 100 couples. Individual differences in partner A’s negative affect were positive predictors of partner B’s interpersonal calming, and individual differences in partner A’s expressive suppression were negative predictors of partner B’s interpersonal calming. Further, individual differences in male partners’ expressivity were significant positive predictors of female partners’ interpersonal calming, and individual differences in male partners’ reappraisal were significant positive predictors of female partners’ interpersonal alerting. These findings suggest that interpersonal worry regulation relates to partners’ expression and intrapersonal regulation of worry, but not equally for men and women.

Although worrying is often a private activity, it can also affect those close to us (Parkinson & Simons, 2012). Expressing worry to others can make them more worried, and suppressing the expression of worry may alleviate this interpersonal effect. Thus, we can potentially regulate someone else’s worry by regulating our own experience and expression of worry-related emotions. The two studies presented in this paper focus on romantic partners’ regulatory responses to each other’s expressed worry about shared concerns. The central idea is that each partner’s interpersonal worry regulation is attuned to the other partner’s expressed emotions.

## Interpersonal Worry Regulation

Social functional accounts propose that a central purpose of emotion expression is to influence other people’s behavior (e.g., Keltner & Haidt, 1999; Parkinson, 1996; Van Kleef, Van Doorn, Heerdink, & Koning, 2011). One process underlying such interpersonal influence is social referencing (Feinman, 1982), whereby one person’s expressed emotions modify another person’s interpretations and evaluations of events. For example, calm expressions can reduce other people’s alertness to potential risks leading to less cautious responses (Latané & Darley, 1968), whereas anxious expressions can increase other people’s alertness to potential risks leading to more cautious responses (Parkinson, Phiri, & Simons, 2012; Parkinson & Simons, 2009; Sorce, Emde, Campos, & Klinnert, 1985). Extending these findings, the present research investigates the idea that romantic partners regulate their expression of worry (i.e., concern-directed anxiety, Parkinson & Simons, 2012) in order to achieve social referencing effects of this kind. In other words, partners regulate their own emotions (intrapersonal emotion regulation) as a way of regulating the emotions experienced by their partners (interpersonal emotion regulation, Zaki & Williams, 2013). For example, we may communicate calm in order to reassure our partner that a concern is not as serious as it may seem (*interpersonal calming*, Parkinson & Simons, 2012), or we may express worry in order to draw our underresponsive partner’s attention to potential concerns (*interpersonal alerting*; Parkinson & Simons, 2012, see also Ein-Dor & Tal, 2012).

Interpersonal calming is intended to provide partners with emotional support and may alleviate their negative affect by reducing stress appraisals or enhancing appraisals of coping potential (e.g., Cohen & Wills, 1985). By contrast, interpersonal alerting may result in our partners feeling worse rather than better, thus providing an interesting example of the underinvestigated phenomenon of downward interpersonal emotion regulation (e.g., Niven, Totterdell, & Holman, 2009). According to Tamir, Ford, and Gilliam (2013), downward emotion regulation is generally motivated by instrumental rather than hedonistic goals. This conclusion may apply to interpersonal emotion regulation in addition to the intrapersonal emotion regulation originally considered by Tamir and colleagues (2013). For example, recent research by Netzer, Van Kleef, and Tamir (2015) showed that participants wanted allies to feel more angry prior to an aggressive computer game in order to maximize their own rewards. Instrumental goals may play a similar role in the kinds of interpersonal worry regulation investigated by the present research. For example, one reason for alerting romantic partners to problems is to recruit their help in coping with those problems. Before this can happen, we need to increase partners’ worry levels to the extent that they are appropriately concerned about the currrent issue.

## Interpersonal Predictors of Calming and Alerting

What leads romantic partners to engage in interpersonal calming or alerting? We propose that the motivation to regulate our partner’s worry and the implementation of partner-directed regulation (i.e., calming or alerting) depend to a large extent on how our partner experiences, expresses, and regulates his or her own worry. When our partner expresses undue worry (or refrains from suppressing expression of worry, e.g., Gross & John, 2003), we are often motivated to offer reassurance by engaging in interpersonal calming. Conversely, when our partner seems unduly unconcerned about what is happening (e.g., because he or she is reappraising concerns in a more positive light, Gross & John, 2003), we are often motivated to engage in interpersonal alerting in order to get him or her to take matters more seriously.

The present research assessed whether one partner’s (partner A’s) worry-related emotions and the intrapersonal regulatory processes that affect these emotions and their expression (e.g., Gross & John, 2003) predict the other partner’s (partner B’s) interpersonal worry regulation (i.e., calming and alerting). Because interpersonal calming is intended to alleviate another person’s concerns, it should be more common when our partner is more worried and expresses more worry. Correspondingly, it should be less common when our partner suppresses worry expressions (Srivastava, Tamir, McGonigal, John, & Gross, 2009) or minimizes the significance of concerns by using reappraisal (Gross & John, 2003). By contrast, because alerting is intended to emphasize the seriousness of concerns, it should be more common when our partner is less worried, expresses less worry, or seems to be underestimating current threat levels (i.e., reappraising concerns to a greater extent).

Based on this reasoning, Study 1 assessed hypotheses concerning the effects of partner A’s experienced worry on partner B’s real-time interpersonal worry regulation, and Study 2 assessed hypotheses concerning the effects of individual differences in partner A’s experience, expression, and intrapersonal regulation of emotion on partner B’s interpersonal worry-regulation tendencies. The specific hypotheses of Study 1 were that partner A’s experienced worry would be a positive predictor of partner B’s interpersonal calming and motivation to regulate partner A’s worry, and a negative predictor of partner B’s interpersonal alerting. The specific hypotheses of Study 2 were that partner A’s dispositional negative affect (including worry-related anxiety, Clark & Watson, 1991) and expressivity would be positive predictors of partner B’s interpersonal calming and negative predictors of partner B’s interpersonal alerting, and that partner A’s expressive suppression and reappraisal tendencies would be negative predictors of partner B’s interpersonal calming and positive predictors of partner B’s interpersonal alerting.

## Gender and Partner-Directed Regulation

Across both studies, we further hypothesized that gender would moderate relations between partner A’s experience, expression, and intrapersonal regulation of emotion and partner B’s interpersonal worry regulation. Previous research suggests that women’s social support provision is often more sensitive than men’s to partners’ emotional needs (e.g., Cutrona, Shaffer, Wesner, & Gardner, 2007; Neff & Karney, 2005). We predicted that similar gender differences would also apply to interpersonal worry regulation. In particular, we hypothesized that male partners’ experience, expression, and intrapersonal regulation of worry would be stronger predictors of female partners’ alerting and calming attempts than vice versa.

These predictions accord with the idea that women’s greater skill at decoding emotion expressions (e.g., Hall, 1978; Hall & Matsumoto, 2004) partly reflects their greater need to respond to subtle nonverbal signals. This need in turn depends on gender inequalities in access to resources, which leave women with less power to influence outcomes using other means (e.g., Henley, 1977; Keltner, Gruenfeld, & Anderson, 2003). Similarly, men’s socialized tendency to take on more powerful roles in romantic relationships may require female partners to be more attuned to male partners’ expressive displays, including their worry expressions.

## The Present Research

Both of these studies used dyadic designs that involved collecting data from both partners in established heterosexual couples. Study 1 assessed partner A’s real-time expression of worry as a predictor of partner B’s use of interpersonal calming and alerting (and motivation to implement interpersonal worry regulation) during conversations between couples discussing a shared concern. Study 2 assessed partner A’s dispositional negative affect, expressivity, and intrapersonal emotion-regulation style as predictors of partner B’s more enduring interpersonal worry-regulation styles using data from a dyadic survey.

## Study 1

Study 1 investigated quasi-naturalistic conversations between romantic partners about shared concerns using measures collected in video-cued recall procedures (e.g., Gottman & Levenson, 1985) and postconversation questionnaires. We assessed relations between partner A’s worry and partner B’s use of interpersonal worry-regulation strategies (calming and alerting) and motivation to reduce A’s worry. Our central hypothesis was that partner A’s worry would positively predict partner B’s calming attempts and motivation to reduce A’s worry, but negatively predict partner B’s interpersonal alerting attempts. We also expected male partners’ worry to have relatively stronger associations with female partners’ interpersonal worry regulation in line with previous findings concerning gender and the provision of sensitive social support (e.g., Cutrona et al., 2007; Neff & Karney, 2005).

### Method

#### Participants

We recruited 40 heterosexual couples by posting Internet advertisements and distributing leaflets around university and public locations.^1^ Participants’ ages ranged from 18 to 57 years (*M* = 25.65, *SD* = 6.51). None of the participants reported suffering from any mental health problems, and all had been in their current relationship for at least 6 months (*M* = 36.81 months, *SD* = 32.03). Thirty-five of the couples were living together at least some of the time, including 8 married couples and 17 fully cohabiting couples. Participants’ scores on the relationship depth (*M* = 3.56, *SD* = 0.27), social support (*M* = 3.55, *SD* = 0.36), and conflict (*M* = 1.91, *SD* = 0.52) subscales of Pierce, Sarason, and Sarason’s (1991) Quality of Relationships Inventory (QRI, scored from 1–4) administered online approximately 1 week before the study indicated high relationship quality. Participating couples received £40 (≈ $60) for their time and effort.

#### Procedure

On arrival, each couple spent a few minutes deciding which worrying issue currently affecting them both (i.e., shared concerns) they would be prepared to discuss during subsequent videotaped conversations. They were encouraged to choose topics that had not been discussed in great detail and continued to be relevant. Selected discussion topics covered a range of issues including where to stay after an upcoming party, plans for summer, cooking habits, managing time apart, financial worries, and moving house. Participants’ prediscussion ratings (on 7-point scales running from 1 *not at all* to 7 *extremely*) of the importance of their discussed concerns (*M* = 5.50, *SD* = 0.80) and of how worried they were about these concerns (*M* = 4.60, *SD* = 0.92) confirmed their continuing emotional relevance.^2^

Partners were seated in comfortable chairs facing each other across a small coffee table. They then conversed as naturally as possible^3^ about the worrying topic for 4 min. Participants’ facial expressions during the conversation were video-recorded using two tripod mounted digital 8-mm color cameras, connected to a DataVideo SE800 Digital Video Switcher, which combined the video signals into a split-screen recording showing both partners’ faces.

After 4 min of conversation, one researcher took each partner to a separate cubicle to complete questionnaires. Meanwhile a second researcher edited a 2.5-min video segment from the conversation starting approximately 1 min into the recording.^4^ After completing their questionnaires, participants individually completed two video-cued recall tasks (following procedures documented by Simons & Parkinson, 2009). In Task 1, participants continuously rated their level of worry during the original conversation while viewing the video recording. In Task 2, the same video-recording was paused at 10-s intervals, and participants made interval-contingent ratings of their motivation to regulate their partner’s worry. Finally, we debriefed participants and asked for signed consent to use the video data.

### Measures

#### Worry

Participants provided continuous ratings of their worry level in the first video-cued recall task using the Continuous Measurement System (CMS; Messinger, Mahoor, Chow, & Cohn, 2009), which displayed a mouse-controlled continuous rating scale on the computer monitor alongside the video recording of the interaction. Worry ratings scored on a 100-point scale (−50 *not at all worried* to +50 *extremely worried*) were recorded for every frame of the video presentation. We calculated aggregated ratings using mean scores across 1-s bins, with missing data replaced using imputation where appropriate.

Two additional measures were used to supplement self-ratings of worry. First, participants continuously rated their partner’s worry in a separate viewing of the videotape. Second, one independent observer provided a general rating of each partner’s level of expressed worry during the same videotaped conversation, and a second independent observer performed the same rating procedure for 50% of the conversations (the two coders’ ratings correlated at *r*(40) = .65, *p* < .001). The 7-point rating scale for this coding task used the single item “How much worry was expressed on the participant’s face?” Aggregated self-rated worry was reliably positively correlated with both aggregated partner-rated worry, *r*(80) = .40, *p* < .001, and observer-coded expressed worry, *r*(80) = .27, *p* = .016.

#### Interpersonal alerting and calming

Immediately after the conversation, participants individually completed an adapted online version of the Interaction Rating Scale (IRS; Simons, Pasqualini, Reddy, & Wood, 2004), which included single-item measures of interpersonal calming (“To what extent were you trying to get your partner to feel calmer about this issue?”) and alerting (“To what extent were you trying to get your partner to appreciate how worrying this issue is?”). Participants rated both items on 7-point scales running from *not at all* (1) to *very much* (7). To validate these self-ratings, we used corresponding items assessing partners’ interpersonal alerting and calming (“To what extent was your partner trying to get you to . . .?”). Ratings by self and partner of participants’ interpersonal alerting and calming correlated positively for alerting, *r*(80) = .31, *p* = .005, and marginally positively for calming, *r*(80) = .19, *p* < .093, supporting the validity of the self-ratings.

#### Motivation to regulate partner’s worry

Participants rated their motivation to regulate their partner’s worry every 10 s during the second video-cued recall task in response to the following question: “To what extent did you want your partner to feel less worried during this clip?” The 7-point response scale ran from 1 (*not at all*) to 7 (*completely*).^5^

#### Analysis procedure

Because of interpartner dependencies in our data, we assessed effects using Statistical Package for the Social Sciences (SPSS)’s mixed models procedure for multilevel modeling following the principles of Kenny, Kashy, and Cook’s (2006) Actor-Partner Interdependence Model treats participants (partners) as nested within dyads and assesses statistically independent actor and partner effects. In APIM terminology, an “actor effect” means that a participant-related predictor influences an outcome relating to that same participant (*intra*personal effect) whereas a “partner effect” means that a participant-related predictor influences an outcome relating to the other participant in the same dyad (*inter*personal effect). The focus of the present research is on assessing interpersonal (partner) effects that are presented below.^6^

**Figure 1 fig1:**
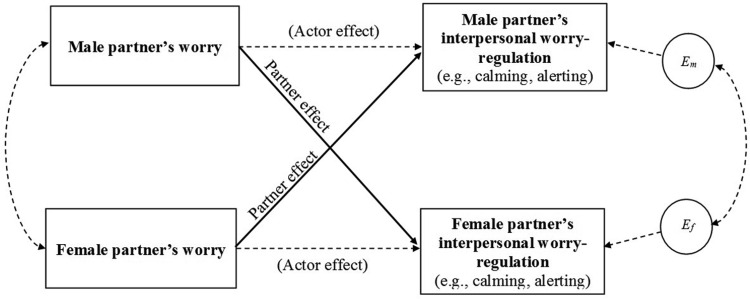
Model of actor and partner effects for Study 1 based on the Actor-Partner Interdependence Model (APIM) (Kenny et al., 2006). *E*_*m*_ and *E*_*f*_ denote error terms for male and female outcomes, respectively. Solid arrows indicate paths of direct interest to the present research.

Gender was used to distinguish partners in each couple. All analyses assessed whether gender moderated fixed actor and partner effects of self-ratings of worry on motivation to regulate partner’s worry, alerting, and calming. Where there were significant or near-significant interactions with gender, we calculated actor and partner effects separately for males and females using dummy codes for each gender in a two-intercept model (see Kenny, Kashy, & Cook, 2006). All variables were grand-mean centered prior to analysis. All analyses in studies 1 and 2 were also run using relationship duration as a control variable, but the same significant effects were obtained in every case. See Figure 1 for a diagram depicting the effects tested by the APIM model used in Study 1.[Fig-anchor fig1]

### Results

#### Descriptive statistics and correlations

Table 1 presents means, standard deviations, and within-dyad (i.e., interpartner) correlations for worry, calming, alerting, and motivation to regulate partner’s worry. Paired samples *t* tests revealed no significant gender differences on any of these measures. Pearson’s correlations revealed no significant associations between male and female partners’ worry scores, or between their scores on motivation to regulate partner’s worry, calming, or alerting. Table 2 presents within-dyad (interpartner) correlations between worry and the three interpersonal worry-regulation measures (corresponding to the partner effects from the APIM analyses reported below).[Table-anchor tbl1][Table-anchor tbl2]

#### Interpersonal effects of worry on calming and alerting

APIM analyses revealed that partner A’s worry was a significant positive predictor of partner B’s interpersonal calming, *t*(64.16) = 1.72, *p* = .045. In other words, participants reported trying to calm their partners more when those partners reported higher levels of worry. However, partner A’s worry did not reliably predict partner B’s alerting across the sample as a whole.

#### Interpersonal effects on motivation to regulate partner’s worry

The video-cued recall data also allowed us to assess the overtime effect of partner A’s worry on partner B’s motivation to regulate A’s worry. We first computed binned scores for each 10-s period of the continuous worry ratings to correspond to the intervals at which motivation to regulate partner’s worry was assessed. We subjected the resulting data to multilevel modeling with time-points nested within dyads following the principles of overtime analysis detailed by Kenny et al. (2006). Controlling for gender and time-point, partner A’s worry was a significant positive predictor of partner B’s motivation to regulate A’s worry, *t*(33.73) = 2.14, *p* = .020.

#### Moderation by gender

There were marginally significant interactions between gender and the effect of partner A’s worry on partner B’s calming, *t*(61.47) = 1.29, *p* = .101, and alerting, *t*(69.75) = 1.35, *p* = .091. Males’ worry was a significant positive predictor of female partners’ calming, *t*(37) = 2.72, *p* = .005, but females’ worry was not a significant predictor of male partners’ calming, *t*(37) = 0.25, *p* = .404, and males’ worry was a significant negative predictor of female partners’ alerting, *t*(37) = 2.09, *p* = .022, but females’ worry was not a significant predictor of male partners’ alerting, *t*(37) = 0.03, *p* = .487.

The overtime effect of partner A’s worry on partner B’s motivation to regulate A’s worry also interacted significantly with gender, *t*(1,184.00) = 2.87, *p* = .002. Male partners’ worry had a stronger effect on female partners’ worry-reduction motivation, *t*(15.26) = 2.44, *p* = .014, than female partners’ worry had on male partners’ worry-reduction motivation, *t*(26.31) = 1.58, *p* = .063.

### Discussion

Study 1 investigated interpersonal worry regulation in romantic couples during real-time conversations about a current concern. As hypothesized, partner A’s worry positively predicted partner B’s interpersonal calming attempts and motivation to reduce A’s worry. Although partner A’s worry did not significantly predict partner B’s alerting across both genders, male participants’ worry was a significant negative predictor of female partners’ alerting.

Gender also significantly moderated the predictive effect of partner A’s worry on partner B’s motivation to reduce A’s worry, showing that changes in male partners’ worry during the conversation were relatively stronger predictors of females’ motivation to reduce partners’ worry. Similarly, male partners’ worry was a significant positive predictor of female partners’ calming, but female partners’ worry was not a significant predictor of male partners’ calming. These findings are consistent with our proposal that women are more attuned than men to their partners’ changing worry levels, in a way that might prepare them for interpersonal regulation including sensitive provision of social support (e.g., Cutrona et al., 2007; Neff & Karney, 2005).

## Study 2

Study 2 was a dyadic survey of a larger sample of heterosexual couples designed to assess whether individual differences in partner A’s experience, expression, and regulation of emotion predicted partner B’s tendencies to engage in interpersonal calming and alerting. Unlike Study 1, which focused on real-time interpersonal worry regulation, Study 2 collected participants’ reports of their typical use of calming and alerting when discussing shared concerns with their partners. The increased sample size also permitted refinement of scales to assess our key constructs by extending and adapting Study 1’s single-item calming and alerting measures.

Study 1 confirmed our hypotheses that partner A’s worry would predict partner B’s interpersonal worry-regulation attempts and motivation to regulate A’s worry. Study 2 assessed whether partner A’s dispositional negative affect (which relates to anxiety, e.g., Clark & Watson, 1991) has comparable predictive effects on partner B’s habitual calming and alerting when discussing shared concerns. We hypothesized that partners of participants with higher levels of negative affect (and therefore greater tendencies to experience worry) would use more calming intended to make their partners less worried, and less alerting intended to make their partners appreciate the seriousness of concerns.

In Study 2, we also assessed individual differences in expressivity (Gross & John, 1995, 1997) and intrapersonal regulation tendencies (Gross & John, 2003) as predictors of partners’ interpersonal calming and alerting. Because interpersonal worry regulation is intended to modify partners’ worry levels, it should be responsive to information about those worry levels available from partners’ regulated or unregulated verbal, facial, and bodily expressions. Partners who provide clearer worry signals either because of their characteristically higher expressivity or their lower levels of expressive suppression (Gross & John, 2003) should therefore solicit more calming and less alerting.

People regulate antecedents of emotion in addition to its expressive consequences (Gross & John, 2003). The most investigated antecedent-focused strategy, reappraisal, involves interpreting emotional events in a different light in order to reduce their emotional impact. In other words, high-reappraisers tend to minimize the emotional significance of potential concerns. Partners of high-reappraisers may therefore react by attempting to emphasize the seriousness of their worries. For these reasons, we predicted that partners of participants scoring higher on reappraisal would use more alerting and less calming.

As in Study 1, we also hypothesized that gender would moderate these interpersonal effects. In particular, women’s greater delivery of sensitive social support (e.g., Neff & Karney, 2005) should mean that their interpersonal calming and alerting are more strongly predicted by partners’ negative affect, expressivity, expressive suppression, and reappraisal.

### Method

#### Participants

We recruited 106 heterosexual couples using local contacts, advertisements, and online announcements. Each couple was paid £20 (≈ $30) for participating. Six couples were excluded due to missing data, leaving a sample of 100 couples aged between 18 and 60 years (*M* = 27.36, *SD* = 7.55), who had been in a relationship for at least 6 months (*M* = 61.01 months, *SD* = 65.60). Sixty-six of the couples lived together on a permanent basis. Quality of Relationships Index (QRI; Pierce, Sarason, & Sarason, 1991) scores (depth *M* = 3.51, *SD* = 0.42; social support *M* = 3.45, *SD* = 0.45; conflict *M* = 1.94, *SD* = 0.52) indicated high relationship quality.

#### Procedure

Couples signed up after reading an e-mailed information letter. Once both partners had agreed to take part, they were sent a web-link to an online informed consent procedure and survey. Each couple member was asked to complete the survey independently of their partner. Computer-based data entry prevented partners from directly comparing answers.

### Measures

#### Negative affect

Participants reported the level of negative affect (NA) experienced during the past 2 weeks using seven items from the Positive and Negative Affect Scales (PANAS; Watson, Clark, & Tellegen, 1988) rated on 5-point scales running from *very slightly or not at all* (1) to *extremely* (5). Reliability was satisfactory (α = .76).

#### Berkeley Expressivity Questionnaire

We used Gross and John’s (1995, 1997) 16-item Berkeley Expressivity Questionnaire (BEQ) to assess emotional expressivity. Participants rated items on 7-point scales running from *strongly disagree* (1) to *strongly agree* (7). Overall scores were computed by averaging means across three subscales, each of which had satisfactory reliability in the present study (αs > .70). Sample items include: “I am an emotionally expressive person” and “What I am feeling is written all over my face.”

#### Emotion Regulation Questionnaire

Gross and John’s (2003) 10-item Emotion Regulation Questionnaire (ERQ) assesses individual differences in tendencies to use expressive suppression (α = .81) and cognitive reappraisal (α = .77) as intrapersonal emotion-regulation strategies. Participants rated items on 7-point scales running from *strongly disagree* (1) to *strongly agree* (7). Sample items include “I control my emotions by not expressing them” (expressive suppression) and “I control my emotions by changing the way I think about the situation I am in” (reappraisal).

#### Calming and alerting

We developed scales assessing calming and alerting specifically for use in the present study. Participants rated how they typically dealt with a shared concern or worry when discussing it with their partner. Four items assessed partner-directed calming tendencies, including an adapted version of Study 1’s calming item (“I try to calm my partner down”), as well as items focusing on related aspects of expressing calm during conversations about shared concerns with partners (e.g., “I try to act calm and composed”). Another 4 items assessed partner-directed alerting tendencies including an adapted version of Study 1’s alerting item (“I try to make my partner see how worrying the issue really is”), as well as other items focusing on related aspects of expressing worry during conversations with partners about shared concerns (e.g., “I try to show how worried I am”). For validation purposes, corresponding items also assessed participants’ perceptions of their partner’s calming and alerting tendencies. All items were rated on 5-point scales (1 = *not at all like us* to 5 = *a great deal like us*). Cronbach’s alphas for the 4-item calming and alerting scales were satisfactory (> .70). We also obtained significant positive correlations between scores on self-rated and other-rated scales for both calming, *r*(200) = .53, *p* < .001, and alerting, *r*(200) = .49, *p* < .001, supporting the validity of our self-rated measures.

### Results

#### Descriptive statistics, within-dyad correlations, and gender differences

**Figure 2 fig2:**
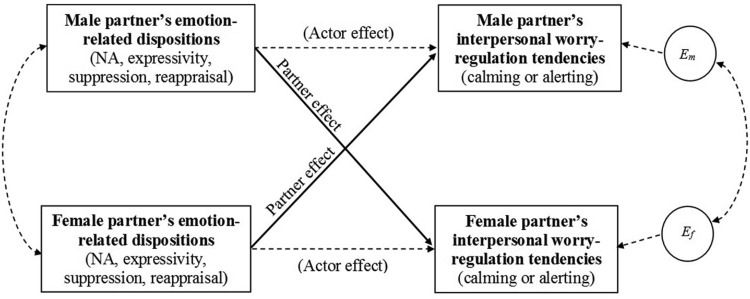
Model of actor and partner effects for Study 2 based on the Actor-Partner Interdependence Model (APIM) (Kenny et al., 2006). *E*_*m*_ and *E*_*f*_ denote error terms for male and female outcomes, respectively. Solid arrows indicate paths of direct interest to the present research. NA = negative affect.

Descriptive statistics for the individual difference predictors and regulatory outcomes assessed in Study 2 are included in Table 1. Paired samples (within-dyad) *t* tests revealed significant gender differences in both interpersonal alerting and calming tendencies. Calming was higher for male partners than for female partners, whereas alerting was higher for female partners than for male partners. Males were also significantly lower than females on expressivity, and significantly higher than females on suppression, but the gender differences in calming and alerting remained significant after controlling for each of these variables. There were no significant gender differences in reappraisal or NA scores. As in Study 1, Pearson’s correlations revealed no statistically reliable within-dyad (i.e., interpartner) associations between male and female partners’ scores on matching variables. Table 3 presents interpartner (within-dyad) correlations between the individual-difference predictors and (partners’) interpersonal worry-regulation outcomes (calming and alerting) corresponding to the APIM partner effects reported below. Figure 2 depicts the model of actor and partner effects used in Study 2.[Table-anchor tbl3][Fig-anchor fig2]

#### Effects on partner’s calming and alerting

Partner effects from APIM analyses (following the same procedures as Study 1) allowed us to test our hypotheses about the effects of partner A’s individual differences on partner B’s use of calming and alerting.^7^ Partner A’s suppression was a significant negative predictor of partner B’s calming, *t*(185.56) = 2.70, *p* = .004, whereas partner A’s NA, *t*(160.77) = 1.81, *p* = .036, and expressivity, *t*(187.94) = 1.77, *p* = .039, were significant positive predictors of partner B’s calming. Partner A’s reappraisal was a significant positive predictor of partner B’s alerting, *t*(187.71) = 1.73, *p* = .043. Thus, partners of participants who were more likely to suppress their emotion expressions used less calming, partners of participants higher in negative affect or in expressivity used more calming, and partners of participants who were more likely to reappraise their concerns used more alerting.

#### Moderation by gender

The effect of partner A’s expressivity on partner B’s calming interacted significantly with gender, *t*(192.21) = 1.68, *p* = .047. Male partners’ expressivity was a significant positive predictor of female partner’s calming, *t*(97) = 2.34, *p* = .011, but female partners’ expressivity was not a significant predictor of male partner’s calming, *t*(97) = 0.08, *p* = .940.

The effect of partner A’s reappraisal on partner B’s alerting showed a marginal interaction with gender, *t*(189.18) = 1.32, *p* = .094. Male partners’ reappraisal was a significant positive predictor of female partner’s alerting, *t*(97) = 2.34, *p* = .011, but female partners’ reappraisal was not a significant predictor of male partner’s alerting, *t*(97) = 0.28, *p* = .777. Gender showed no significant interactions with partner effects of NA or suppression.

### Discussion

Study 1 showed that partner A’s levels of worry during a conversation predicted partner B’s interpersonal worry regulation. Study 2 extended these findings by showing how individual differences in partner A’s experience, expression, and intrapersonal regulation of emotion predicted partner B’s typical use of interpersonal calming and alerting during conversations about shared concerns.

As hypothesized, partner A’s dispositional negative affect was a significant positive predictor of partner B’s use of interpersonal calming. In other words, partners of participants who experience more negative affect used more calming. This finding parallels Study 1’s predictive effects of partner A’s worry on partner B’s concurrent calming attempts, and suggests that these effects reflect more consistent relational patterns. Also as hypothesized, partner A’s expressivity and use of expressive suppression had similar effects on partner B’s use of calming. In other words, the tendency to use calming related not only to partners’ experience of negative affect, but also to the strength of its expression. Participants who had clearer expressions either because of their dispositionally high expressivity or their limited use of suppression apparently solicited greater calming from their partners, thus extending Srivastava et al.’s (2009) previous finding that participants lower in suppression reported higher levels of social support.

Correspondingly, partners of participants with higher reappraisal scores used more alerting. This finding is consistent with alerting’s interpersonal function as a means of encouraging relationship partners to treat concerns with due seriousness rather than trying to minimize their importance. The reported effects of partner A’s suppression and alerting on partner B’s calming and alerting also strongly imply that intrapersonal and interpersonal emotion regulation are interrelated (see also Butler et al., 2003). For example, our findings are consistent with the conclusion that a male partner’s habitual reappraisal may lead his female partner to adopt the corrective interpersonal regulatory strategy of alerting.

Turning to gender, we found that male partners’ expressivity was a positive predictor of female partners’ calming, but there was no corresponding effect of female partners’ expressivity on male partners’ calming. Similarly, male partners’ reappraisal was a positive predictor of female partners’ alerting, but there was no corresponding effect of female partners’ reappraisal on male partners’ alerting. In other words, female partners’ interpersonal worry regulation was relatively more closely related to partners’ style of expressing and regulating emotions as hypothesized. An unpredicted finding was that male partners also reported more calming and less alerting than female partners more generally. These gender differences remained after controlling for other measures on which males and female participants differed (expressivity and suppression). Moreover, there were no corresponding differences in negative affect or reappraisal, making it seem unlikely that gender’s effect on calming and alerting reflected the sex-typed dispositions to experience worry sometimes reported in previous research (e.g., Conway, Wood, Dugas, & Pushkar, 2003; Kulik, 2006). Instead, it seems that people often conform to conventional gender roles when communicating with their heterosexual partners. In particular, masculine roles may encourage male partners to convey their mastery (e.g., Boggiano & Barrett, 1991) over worrying issues by using more calming and by refraining from expressing undue concern. Thus, the division of emotional labor in romantic relationships may partly follow gender-defined lines (cf. Duncombe & Marsden, 1993).

## General Discussion

Findings from these two studies are consistent with the proposal that partner A’s experience, expression, and regulation of worry are predictors of partner B’s interpersonal worry regulation (i.e., calming and alerting). Further, worry-related measures were often stronger predictors of female partners’ than male partners’ interpersonal worry regulation as hypothesized. Both studies used dyadic designs that allowed us to demonstrate associations between measures collected from different partners controlling for intrapersonal (actor) effects. We were also able to validate our new measures using independent ratings by partners or neutral observers, and by demonstrating meaningful interrelations even when data were collected at different times and in different forms (e.g., using questionnaires and video-cued recall procedures).

Our findings also provide novel evidence about the underinvestigated phenomenon of downward interpersonal emotion regulation (Netzer et al., 2015; Niven et al., 2009). In particular, participants’ use of alerting involved trying to make their partners feel more rather than less worried (i.e., worse rather than better). Study 1 showed that female participants used more alerting when their male partners were less worried, and Study 2 showed that female participants used more alerting when their male partners used more reappraisal. These findings extend previous research showing that women are relatively more sensitive to partners’ emotional needs when delivering social support (e.g., Cutrona et al., 2007; Neff & Karney, 2005). In our studies, women were more sensitive to partners’ regulatory style as well as to their emotions, and this sensitivity affected not only their emotional support (i.e., calming) but also their use of downward interpersonal regulation (i.e., alerting). However, our use of an entirely heterosexual sample means that the reported partner effects confound the gender of the interpersonal worry regulator with the gender of the person whose worry is being regulated (regulation target). It is therefore important that future studies assess comparable interpersonal effects across a wider range of relationships including homosexual relationships and relationships between same-gender friends.

A consistent finding across both studies was that partner A’s worry-related emotion positively predicted partner B’s interpersonal calming. However, the effect of partner A’s worry on partner B’s calming in Study 1 was moderated by gender, whereas the effect of partner A’s negative affect on partner B’s calming in Study 2 was not. This apparent difference may suggest that female partners’ interpersonal regulation may be especially sensitive to male partners’ worry during discussions about shared concerns but not to male partners’ negative affect more generally. Alternatively, the task of discussing shared concerns for four minutes may have encouraged Study 1 participants not only to express any worry that they were experiencing but also to reappraise their worrying concerns. Thus, worry’s interpersonal effects in Study 1 may have reflected its enhanced association with expression and reappraisal, both of which had relatively stronger interpersonal effects on female partners’ interpersonal worry regulation in Study 2.

## Limitations and Future Directions

The data presented in this manuscript permit no firm conclusions about causality. For example, the reported association between male partners’ reappraisal and female partners’ interpersonal alerting may either mean that female partners’ alerting is a corrective response to male partners’ reappraisal or that male partners’ reappraisal develops as a strategy for dealing with worrying concerns highlighted by female partners’ alerting. Indeed, these two interpersonal processes may be mutually reinforcing with female partners increasing their use of alerting in order to unsettle male partners’ escalating attempts to reappraise concerns. However, in other cases, the nature of the reported effect makes certain causal explanations less likely. For example, it seems more plausible to suggest that partner B tried to calm partner A in response to A’s expressed worry (or negative affect more generally), than that B’s calming attempts resulted in A becoming more worried (or higher in negative affect). Indeed, to the extent that B’s calming is successful, we would expect it to reduce rather than increase A’s worry and negative affect.

Another possible explanation for those of our findings that are based on cross-sectional data is partner selection. In particular, people may initially select (or stay with) romantic partners who fulfill certain emotional needs. For example, worry-prone individuals may seek out partners who can share worries or who can help to deal with them (e.g., by maintaining a reassuring calm façade). Relatedly, previous research suggests that people select romantic partners partly on the basis of perceived compatibility of attachment styles (e.g., Holmes & Johnson, 2009; Strauss, Morry, & Kito, 2012). However, like reverse causation, partner selection seems an implausible explanation for many of the partner effects reported above. For example, why would male reappraisers choose female partners who consistently use more alerting? It seems more plausible that female partners’ alerting develops in response to male partners’ minimization of the significance of concerns over the course of the relationship. It is also worth noting that the overtime effects of worry reported in Study 1 are not susceptible to explanations based on partner selection because they reflect associations between changes in scores between members of the same couples. Future research should use longitudinal designs to provide definitive evidence that partner B’s interpersonal emotion-regulation styles and strategies become more closely attuned to partner A’s expression and intrapersonal of emotion as relationships develop.

Our studies focused on partners’ responses to shared concerns. A further issue for future research is whether similar processes apply when a concern is faced individually by one partner and does not directly affect the other (e.g., one partner’s worries about a job interview). In most romantic relationships, interdependences between partners mean that even personal concerns represent a form of dyadic stress (e.g., Randall & Bodenmann, 2009; Story & Bradbury, 2004), partly because the potential outcomes often bring consequences for both partners (e.g., financial insecurity caused by a partner’s lack of employment), and partly because concern for the other’s well-being can lead to empathic responses (including interpersonal metaworry, Parkinson & Simons, 2012). In our Study 1, partners often differed in how worried they initially felt about a shared concern, and in how their levels of worry changed over time, so the dynamics of interpersonal worry regulation may not be so different in the case of individual rather than shared concerns.

Our focus on worry rather than other kinds of emotion was partly motivated by the discrepant alerting and comfort-seeking interpersonal functions of worry expression (Parkinson & Simons, 2012), which raise the possibility of mistargeted interpersonal responses. For example, calming a partner who is using worry expressions in order to alert you may increase rather than reduce that partner’s expressed worry. Correspondingly, alerting a partner who is suppressing worry expressions in order to calm you may increase rather than reduce that partner’s expressive suppression. Future research should address whether similar unintended emotional consequences arise from other interpersonally expressed emotions. For example, the intended interpersonal effect of anger expression may often be guilt (e.g., Parkinson, 2001; Parkinson & Illingworth, 2009), yet expressed anger often provokes reciprocated anger from partners. How then do romantic partners regulate their anger expressions to address these interpersonal effects? It seems likely that gender also plays a role in this case. Indeed, recent research by Randall, Post, Reed, and Butler (2013) found gender differences in interpartner emotion dynamics relating to general valence rather than specific emotions such as anger or worry. During cooperative interactions, men’s affect tended to change in the same direction as their female partners’ affect over time (inphase coordination), whereas women’s affect tended to diverge from their male partners’ affect (antiphase coordination). This relational pattern is similar to the closer attunement of women’s calming to male partners’ worry found in Study 1. Application of Randall et al.’s (2013) time-based dyadic methodology to changes in worry and anger would help to establish which effects are specific to each of these emotions.

We hope that our focus on interpersonal emotion regulation in naturalistic conversations between people sharing close relationships will inspire further research that supplements more controlled laboratory investigations of less consequential encounters between strangers (see also Parkinson & Manstead, in press). The increasing availability of methodologies and statistical techniques for analyzing the complex reciprocal dynamics operating in ongoing emotional interactions (e.g., Butler, 2011) present exciting opportunities for extending our understanding of how emotions operate between as well as within people (de Rivera & Grinkis, 1986; Parkinson, 1996). The present studies have identified interpersonal effects of worry expression and regulation on relationship partners that merit further investigation using these more intensive methods. In particular, we hope that future studies will clarify when and how partners are able to respond sensitively and appropriately to each other’s expressed worries. Research of this kind may ultimately lead to the development of relational as well as individual interventions targeted at improving interpersonal worry regulation.

## Figures and Tables

**Table 1 tbl1:** Means, SDs, and Within-Dyad Correlations for Study Variables

	Male partner *M* (*SD*)	Female partner *M* (*SD*)	Within-dyad *r*
Study 1			
Worry	2.81 (18.00)	6.95 (15.47)	.14
Calming	3.88 (2.05)	3.28 (1.75)	−.09
Alerting	3.75 (1.91)	3.77 (1.90)	.15
Motivation to regulate partner’s worry	3.89 (1.32)	3.88 (1.65)	−.19
Study 2			
NA	2.02 (0.67)	2.15 (0.71)	−.04
BEQ	4.32 (0.83)	5.18 (0.78)***	.06
ERQ suppression	3.78 (1.27)	2.82 (1.19)***	.09
ERQ reappraisal	4.78 (0.90)	4.75 (0.89)	.03
Calming	3.95 (0.59)	3.24 (0.72)***	−.12
Alerting	2.60 (0.83)	3.22 (0.74)***	.00
*Note.* The within-dyad *r* column shows correlations between male partners and female partners’ scores on each variable (e.g., male worry correlated with female worry). None of these correlations were statistically significant. NA = negative affect; BEQ = Berkeley Expressivity Questionnaire; ERQ = Emotion Regulation Questionnaire.			
*** Significant gender difference. *p* < .001.			

**Table 2 tbl2:** Within-Dyad Correlations Between Partner’s Interpersonal Regulation Measures and Own Worry in Study 1

	Male worry	Female worry	All participants’ worry
Calming	.37*	.06	.23*
Alerting	−.23	.07	−.09
Motivation to regulate partner’s worry	.47**	.26	.38**
*Note*. Male coefficients reflect partner effects on male outcome variables, and female coefficients reflect partner effects on female outcome variables.			
* *p* < .05. ** *p* < .01.			

**Table 3 tbl3:** Within-Dyad Correlations Between Predictors and Partners’ Interpersonal Worry-Regulation Outcomes (Calming and Alerting) in Study 2

	Calming			Alerting		
	Males	Females	All	Males	Females	All
NA	.18	.10	.16*	−.03	.08	−.01
BEQ	.00	.22*	.32**	.10	.05	−.12
ERQ suppression	−.14	−.23*	−.33**	.13	.10	.24**
ERQ reappraisal	−.11	−.07	−.09	.02	.23*	.12
*Note*. Male coefficients reflect partner effects on male outcome variables, and female coefficients reflect partner effects on female outcome variables. NA = negative affect; BEQ = Berkeley Expressivity Questionnaire; ERQ = Emotion Regulation Questionnaire.						
* *p* < .05. ** *p* < .01.						

## Footnotes

1 Data from 2 additional couples were discarded due to computer problems and missing data, and another 20 couples failed to attend their scheduled sessions.

2 There were no significant gender differences in participants’ pre-study ratings of issue importance (male *M* = 5.40, *SD* = 1.50; female *M* = 5.95, *SD* = 1.26) or worry (male *M* = 4.43, *SD* = 1.39; female *M* = 4.88, *SD* = 1.40).

3 Participants’ mean rating of how similar the laboratory conversation was to conversations they normally had with their partner on a 7-point scale running from *not at all* (1) to *very much* (7) was 5.39 (*SD* = 1.21). Participants also discussed a second concern, focusing specifically on its negative aspects, but data from this negative-focus condition are not reported here because they do not relate directly to this paper’s central topic. Analyses assessing effects of conversation order (i.e., whether the negative-focus condition preceded or followed the unmanipulated condition) revealed no significant effects.

4 We made the decision to use only 2.5 min of the 4-min videotape following previous experiences of using video-cued recall in similar tasks. Participants often take up to a minute to familiarize themselves with the unusual setting and the presence of video cameras, so we excluded the first 60 s of the videotape. Further, some couples started to conclude their discussions before 4 min were over, so we excluded the final 30 s.

5 We devised the item assessing motivation to regulate partner’s worry because pilot research using similar video-cued recall procedures suggested that participants were unwilling to acknowledge strategic interpersonal regulation attempts when rating specific moments from an ongoing interaction. However, their video-cued reports of the motivation behind calming and alerting attempts showed meaningful variation over time, and correlated positively with post-conversation measures of related variables.

6 APIM deals with interdependent dyadic data by computing partner effects after controlling for actor effects. We summarize these actor effects here for the sake of completeness. The effect of worry on own calming interacted significantly with gender, *t*(68.82) = 1.68, *p* = .049, and was reliably negative only for females, *t*(37) = 1.69, *p* = .049. In other words, only female participants who were more worried reported less interpersonal calming. Worry was a significant positive predictor of own alerting, *t*(72.13) = 4.77, *p* < .001, and this effect did not interact significantly with gender. In other words, participants engaged in more interpersonal alerting when they felt relatively more worried.

7 As with Study 1, actor effects from the APIM analyses are not centrally relevant to the present paper but are summarized here for the sake of completeness. Both NA and BEQ were reliable negative predictors of own calming, NA: *t*(189.76) = 2.67, *p* = .004; BEQ: *t*(180.99) = 2.00, *p* = .024, and reliable positive predictors of own alerting, NA: *t*(188.86) = 2.47, *p* = .007; BEQ: *t*(191.72) = 2.47, *p* = .007. ERQ reappraisal was a significant negative predictor of own alerting, *t*(191.85) = 1.98, *p* = .025, and a significant positive predictor of own calming, *t*(186.34) = 3.90, *p* < .001. Thus, participants higher in negative affect or expressivity, and participants who were less inclined to reappraise, tended to use less calming and more alerting. Suppression had no significant effects on own calming or alerting.

## References

[c1] Boggiano A., & Barrett M. (1991). Strategies to motivate helpless and mastery-oriented children: The effect of gender-based expectancies. Sex Roles, 25, 487–510. 10.1007/BF00290059

[c2] Butler E. A. (2011). Temporal interpersonal emotion systems: The “TIES” that form relationships. Personality and Social Psychology Review, 15, 367–393. 10.1177/1088868311411164 21693670

[c3] Butler E. A., Egloff B., Wilhelm F. H., Smith N. C., Erickson E. A., & Gross J. J. (2003). The social consequences of expressive suppression. Emotion, 3, 48–67. 10.1037/1528-3542.3.1.48 12899316

[c5] Clark L. A., & Watson D. (1991). Tripartite model of anxiety and depression: Psychometric evidence and taxonomic implications. Journal of Abnormal Psychology, 100, 316–336. 10.1037/0021-843X.100.3.316 1918611

[c6] Cohen S., & Wills T. A. (1985). Stress, social support, and the buffering hypothesis. Psychological Bulletin, 98, 310–357. 10.1037/0033-2909.98.2.310 3901065

[c7] Conway M., Wood W. J., Dugas M., & Pushkar D. (2003). Are women perceived as worrying more than men? A status interpretation. Sex Roles, 49, 1–10. 10.1023/A:1023901417591

[c8] Cutrona C. E., Shaffer P. A., Wesner K. A., & Gardner K. A. (2007). Optimally matching support and perceived spousal sensitivity. Journal of Family Psychology, 21, 754–758. 10.1037/0893-3200.21.4.754 18179347

[c9] de Rivera J., & Grinkis C. (1986). Emotions as social relationships. Motivation and Emotion, 10, 351–369. 10.1007/BF00992109

[c10] Duncombe J., & Marsden D. (1993). Love and intimacy: The gender division of emotion and “emotion work.” Sociology, 27, 221–241. 10.1177/0038038593027002003

[c11] Ein-Dor T., & Tal O. (2012). Scared saviors: Evidence that people high in attachment anxiety are more effective at alerting others to threat. European Journal of Social Psychology, 42, 667–671. 10.1002/ejsp.1895

[c12] Feinman S. (1982). Social referencing in infancy. Merrill-Palmer Quarterly, 28, 445–470.

[c13] Gottman J. M., & Levenson R. W. (1985). A valid procedure for obtaining self-report of affect in marital interaction. Journal of Consulting and Clinical Psychology, 53, 151–160. 10.1037/0022-006X.53.2.151 3998244

[c14] Gross J. J., & John O. P. (1995). Facets of emotional expressivity: Three self-report factors and their correlates. Personality and Individual Differences, 19, 555–568. 10.1016/0191-8869(95)00055-B

[c15] Gross J. J., & John O. P. (1997). Revealing feelings: Facets of emotional expressivity in self-reports, peer ratings, and behavior. Journal of Personality and Social Psychology, 72, 435–448. 10.1037/0022-3514.72.2.435 9107009

[c16] Gross J. J., & John O. P. (2003). Individual differences in two emotion regulation processes: Implications for affect, relationships, and well-being. Journal of Personality and Social Psychology, 85, 348–362. 10.1037/0022-3514.85.2.348 12916575

[c17] Hall J. A. (1978). Gender effects in decoding nonverbal cues. Psychological Bulletin, 85, 845–857. 10.1037/0033-2909.85.4.845

[c18] Hall J. A., & Matsumoto D. (2004). Gender differences in judgments of multiple emotions from facial expressions. Emotion, 4, 201–206. 15222856 10.1037/1528-3542.4.2.201

[c19] Henley N. M. (1977). Body politics: Power, sex, and nonverbal communication. Englewood Cliffs, NJ: Prentice Hall.

[c20] Holmes B. M., & Johnson K. R. (2009). Adult attachment and romantic partner preference: A review. Journal of Social and Personal Relationships, 26, 833–852. 10.1177/0265407509345653

[c21] Keltner D., Gruenfeld D. H., & Anderson C. (2003). Power, approach, and inhibition. Psychological Review, 110, 265–284. 10.1037/0033-295X.110.2.265 12747524

[c22] Keltner D., & Haidt J. (1999). Social functions of emotions at four levels of analysis. Cognition and Emotion, 13, 505–521. 10.1080/026999399379168

[c23] Kenny D. A., Kashy D. A., & Cook W. L. (2006). Dyadic data analysis. New York, NY: Guilford Press.

[c24] Kulik L. (2006). Personality profiles, life satisfaction and gender-role ideology among couples in late adulthood: The Israeli case. Personality and Individual Differences, 40, 317–329. 10.1016/j.paid.2005.06.026

[c25] Latané B., & Darley J. M. (1968). Group inhibition of bystander intervention in emergencies. Journal of Personality and Social Psychology, 10, 215–221. 10.1037/h0026570 5704479

[c26] Messinger D. S., Mahoor M. H., Chow S. M., & Cohn J. F. (2009). Automated measurement of facial expression in infant–mother interaction: A pilot study. Infancy, 14, 285–305. 10.1080/15250000902839963 19885384 PMC2746084

[c27] Neff L. A., & Karney B. R. (2005). Gender differences in social support: A question of skill or responsiveness? Journal of Personality and Social Psychology, 88, 79–90. 10.1037/0022-3514.88.1.79 15631576

[c28] Netzer L., Van Kleef G. A., & Tamir M. (2015). Interpersonal instrumental emotion regulation. Journal of Experimental Social Psychology, 58, 124–135. 10.1016/j.jesp.2015.01.006

[c29] Niven K., Totterdell P., & Holman D. (2009). A classification of controlled interpersonal affect regulation strategies. Emotion, 9, 498–509. 10.1037/a0015962 19653772

[c30] Parkinson B. (1996). Emotions are social. British Journal of Psychology, 87, 663–683. 10.1111/j.2044-8295.1996.tb02615.x 8962482

[c31] Parkinson B. (2001). Anger on and off the road. British Journal of Psychology, 92, 507–526. 10.1348/000712601162310 11534742

[c32] Parkinson B., & Illingworth S. (2009). Guilt in response to blame from others. Cognition and Emotion, 23, 1589–1614. 10.1080/02699930802591594

[c33] Parkinson B., & Manstead A. S. R. (in press). Current emotion research in social psychology: Thinking about emotions and other people. Emotion Review.

[c34] Parkinson B., Phiri N., & Simons G. (2012). Bursting with anxiety: Adult social referencing in an interpersonal balloon analogue risk task (BART). Emotion, 12, 817–826. 10.1037/a0026434 22251046

[c35] Parkinson B., & Simons G. (2009). Affecting others: Social appraisal and emotion contagion in everyday decision making. Personality and Social Psychology Bulletin, 35, 1071–1084. 10.1177/0146167209336611 19474455

[c36] Parkinson B., & Simons G. (2012). Worry spreads: Interpersonal transfer of problem-related anxiety. Cognition and Emotion, 26, 462–479. 10.1080/02699931.2011.651101 22471852

[c37] Pierce G. R., Sarason I. G., & Sarason B. R. (1991). General and relationship-based perceptions of social support: Are two constructs better than one? Journal of Personality and Social Psychology, 61, 1028–1039. 10.1037/0022-3514.61.6.1028 1774625

[c38] Randall A. K., & Bodenmann G. (2009). The role of stress on close relationships and marital satisfaction. Clinical Psychology Review, 29, 105–115. 10.1016/j.cpr.2008.10.004 19167139

[c39] Randall A. K., Post J. H., Reed R. G., & Butler E. A. (2013). Cooperating with your romantic partner: Associations with interpersonal emotion coordination. Journal of Social and Personal Relationships, 30, 1072–1095. 10.1177/0265407513481864

[c40] Simons G., & Parkinson B. (2009). Time-dependent observational and diary methodologies and their use in studies of social referencing and interpersonal emotion regulation. 21st Century Society, 4, 175–186.

[c41] Simons G., Pasqualini M. C., Reddy V., & Wood J. (2004). Emotional and nonemotional facial expressions in people with Parkinson’s disease. Journal of the International Neuropsychological Society, 10, 521–535. 10.1017/S135561770410413X 15327731

[c42] Sorce J. F., Emde R. N., Campos J., & Klinnert M. D. (1985). Maternal emotional signaling: Its effect on the visual cliff behavior of 1 year olds. Developmental Psychology, 21, 195–200. 10.1037/0012-1649.21.1.195

[c43] Srivastava S., Tamir M., McGonigal K. M., John O. P., & Gross J. J. (2009). The social costs of emotional suppression: A prospective study of the transition to college. Journal of Personality and Social Psychology, 96, 883–897. 10.1037/a0014755 19309209 PMC4141473

[c44] Story L. B., & Bradbury T. N. (2004). Understanding marriage and stress: Essential questions and challenges. Clinical Psychology Review, 23, 1139–1162. 10.1016/j.cpr.2003.10.002 14729426

[c45] Strauss C., Morry M. M., & Kito M. (2012). Attachment style and relationship quality: Actual, perceived, and ideal partner matching. Personal Relationships, 19, 14–36. 10.1111/j.1475-6811.2010.01333.x

[c46] Tamir M., Ford B. Q., & Gilliam M. (2013). Evidence for utilitarian motives in emotion regulation. Cognition and Emotion, 27, 483–491. 10.1080/02699931.2012.715079 22917624

[c47] Van Kleef G. A., Van Doorn E. A., Heerdink M. W., & Koning L. F. (2011). Emotion is for influence. European Review of Social Psychology, 22, 114–163. 10.1080/10463283.2011.627192

[c48] Watson D., Clark L. A., & Tellegen A. (1988). Development and validation of brief measures of positive and negative affect: The PANAS scales. Journal of Personality and Social Psychology, 54, 1063–1070. 10.1037/0022-3514.54.6.1063 3397865

[c49] Zaki J., & Williams W. C. (2013). Interpersonal emotion regulation. Emotion, 13, 803–810. 10.1037/a0033839 24098929

